# Activation of K_ATP_ channels in pain modulation: a systematic review of preclinical studies

**DOI:** 10.3389/fphys.2025.1444270

**Published:** 2025-01-29

**Authors:** Signe Schønning Beich, Lili Kokoti, Mohammad Al-Mahdi Al-Karagholi

**Affiliations:** ^1^ Department of Neurology, Danish Headache Center, Rigshospitalet Glostrup, Faculty of Health and Medical Sciences, University of Copenhagen, Copenhagen, Denmark; ^2^ Department of Neurology, Nordsjaellands Hospital-Hilleroed, Hilleroed, Denmark

**Keywords:** nociception, antinociception, ATP sensitive potassium channel, potassium channel, headache

## Abstract

**Objective:**

To systematically review the involvement of K_ATP_ channel activation in pain modulation in preclinical studies.

**Background:**

K_ATP_ channels are expressed at several levels in the spinal and trigeminal pain pathways, where they seem to modulate nociceptive transmission.

**Methods:**

PubMed and Embase databases were searched until 29 January 2024, using the following search string: [(pain) OR (nociception) OR (antinociception) AND (K_ATP_ channel) OR (ATP sensitive potassium channel)]. Non-English and unavailable records, as well as records with non-experimental methodology, were excluded. Inclusion criteria were preclinical studies measuring pain *in vivo* upon activation of the K_ATP_ channel by administering a stimulator or positive modulator. Records were screened based on title and abstract, and those that met the study inclusion criteria were reviewed based on study design, measurements, intervention, and outcomes.

**Results:**

The search resulted in 569 records. In total, 126 duplicates were detected. Subsequently, 438 records were screened by title and abstract, resulting in the exclusion of 396. Based on inclusion criteria, 42 studies were included. The main findings of the present systematic review were that K_ATP_ channel openers can attenuate induced pain in various animal models and potentiate the effects of analgesics.

**Conclusion:**

Local, systemic, spinal, and supraspinal activation of K_ATP_ channels can attenuate pain and potentiate the efficacy of analgesic drugs. One exception was levcromakalim, as the systemic levcromakalim administration, but not a local application, induced pain. This finding is consistent with those of recent human trials. Future studies should investigate the differences in K_ATP_ channel activation between rodents and humans, as well as the differences in activation sites between levcromakalim and other K_ATP_ channel openers.

## 1 Introduction

A large series of preclinical investigations have identified adenosine triphosphate (ATP)-sensitive potassium (K_ATP_) channels as potential targets for novel drugs to treat several pain disorders (Al-Karagholi, 2023). K_ATP_ channels contribute to chemosensory transduction and generation/propagation of action potentials in peripheral nociceptive afferents. These channels are octamer proteins consisting of four pore-forming, inwardly rectifying subunits (Kir family) and four sulfonylurea subunits (SUR family) (Cui et al., 2001; Wu et al., 2011). The Kir subunit identified in K_ATP_ channels is the Kir6 subunit, which is expressed in two isoforms, Kir6.1 and Kir6.2 (Al-Karagholi, 2023). The SUR subunit is expressed in three isoforms: SUR1, SUR2A and SUR2B. The subtypes exist in tissues in different combinations with different properties. Kir6.2/SUR1 are expressed in pancreatic beta cells, trigeminal ganglion, trigeminal nucleus caudalis and central neurons. Kir6.2/SUR2A are found in cardiac and skeletal muscle. Kir6.1/SUR2B and Kir6.2/SUR2B exist in smooth muscle (Al-Karagholi, 2023). Distinct subunit expression has been detected in various cells, including neurons, vascular smooth muscle, and pancreatic beta cells (Babenko et al., 1998). Elevated intracellular ATP reportedly inhibits K_ATP_ channels, leading to reduced potassium efflux and depolarization (Cui et al., 2001; Yamashita et al., 1994). However, the opening of K_ATP_ channels results in hyperpolarization and, thus, a decrease in the neuronal firing frequency (Babenko et al., 1998) and dilation of vascular smooth muscle cells (Christensen et al., 2022). In the current study, we systematically reviewed the implications of K_ATP_ channels in pain transmission following *in vivo* central or peripheral administration of K_ATP_ channel openers in preclinical studies.

## 2 Methods

**FIGURE 1:**
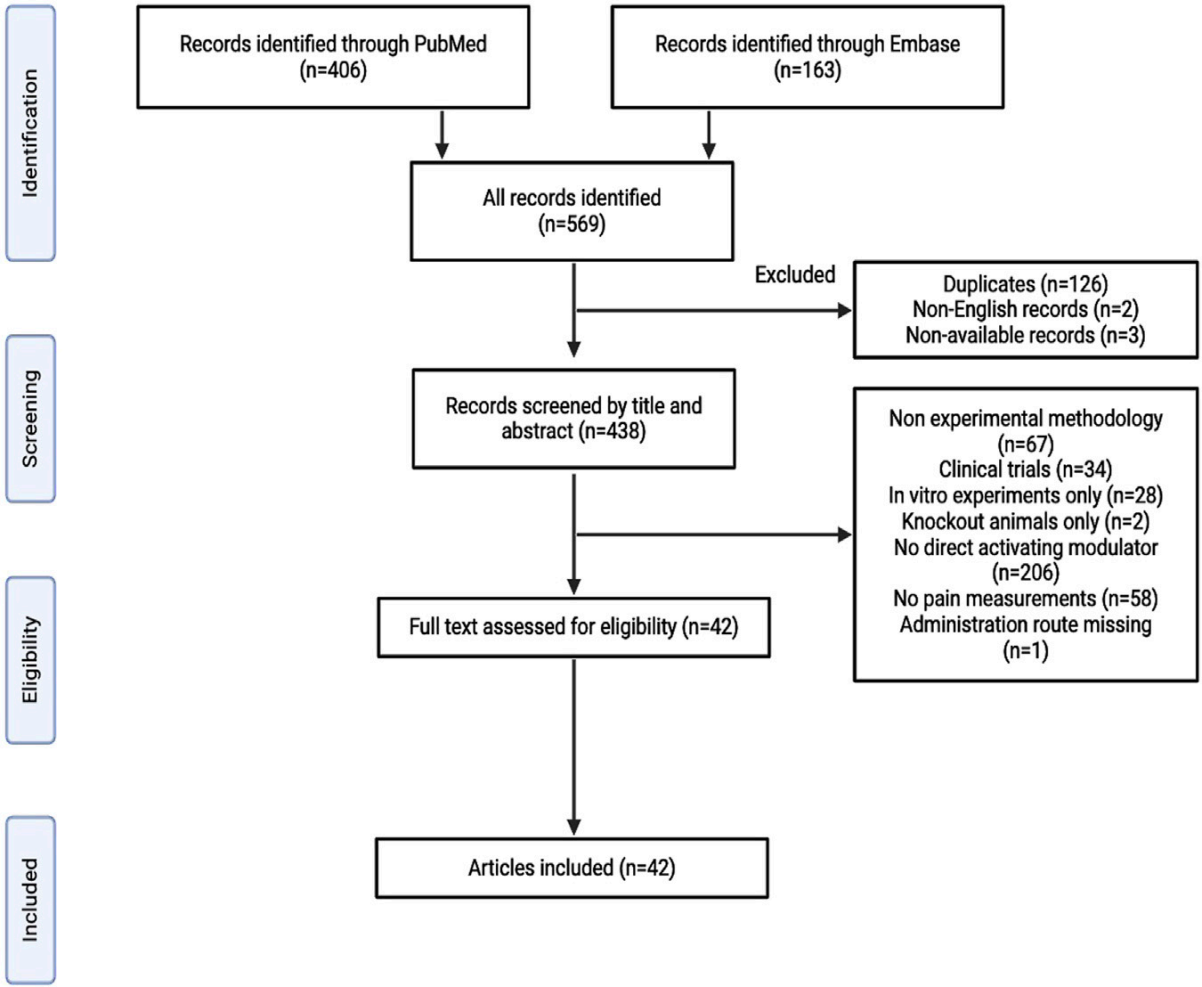
Study flowchart.

A systematic search was performed according to the Preferred Reporting Items for Systematic Reviews and Meta-Analyses (PRISMA) reporting guidelines. PubMed and Embase databases were searched until 29th January 2024, to identify studies that investigated the involvement of K_ATP_ channels in pain transmission using the following search string: [(pain) OR (nociception) OR (antinociception) AND (K_ATP_ channel) OR (ATP-sensitive potassium channel)]. Non-English records and those that could not be retrieved were excluded from the study. In addition, records using non-experimental methods (reviews, conference abstracts, case reports, and meta-analyses) were excluded. The full text was assessed if the title and abstract did not provide all the necessary information. We included *in vivo* preclinical studies exploring pain mechanisms upon the administration of a stimulator or a direct activating modulator of K_ATP_ channels using pain measurement techniques (Figure 1). Data regarding authors, year of publication, study design, measurements, intervention, and outcomes were collected. For the study design, an overview of the experimental protocol was obtained. Pain was measured, and interventions were performed using K_ATP_ channel openers. Data on experiments and outcomes beyond the scope of this review were not evaluated. Data were independently extracted by two investigators (SSB and MMA). Discrepancies were resolved through discussion between the two investigators.

## 3 Results

In total, 569 records were retrieved following the database search, of which 126 were identified as duplicates. Two non-English and three unavailable records were excluded. The remaining 438 articles were screened based on their titles and abstracts. Of these, 396 studies were excluded for the following reasons: non-experimental methodology, clinical trials, only *in vitro* experiments, only knockout animals used, no activating modulator employed, no pain measurements performed, or missing data on administration routes (Figure 1). The remaining 42 studies were included in the final analysis. Several K_ATP_ channel openers (KCO), such as pinacidil, diazoxide, cromakalim, levcromakalim, and NN414, have been used to examine the role of K_ATP_ channels in pain transmission (Table 1). The findings of the included studies are summarized in Tables 2–9.

**TABLE 1:** K_ATP_ channel openers.

K_ATP_ channel openers (KCO)	Action on K_ATP_ channel	Tissue specific^a^	The rout of administration^b^	Crossing the blood–brain barrier (BBB)	Clinical use
Cromakalim (Mannhold, 2004)	SUR2-selective KCO with a greater potency for SURB than for SURA	Smooth muscle cells	Peroral and intravenous administration	Yet to be clarified	Not approved for clinical use
Levcromakalim (Mannhold, 2004)	SUR2-selective KCO with a greater potency for SURB than for SURA	Smooth muscle cells	Peroral administration was investigated in the treatment of asthma and essential hypertension. Intravenous administration dilated cerebral arteries	Given the dilation of cerebral arteries, levcromakalim might cross the BBB	Not approved for clinical use
Pinacidil (Mannhold, 2004)	SUR2-selective KCO with a greater potency for SURB than for SURA	Smooth muscle cells	Peroral administration was investigated in the treatment of essential hypertension	Yet to be clarified	Not approved for clinical use
Diazoxide (Mannhold, 2004)	The binding site remains to be fully elucidated. Several studies indicate that diazoxide is a nonselective SUR agonist	Cardiac, skeletal and smooth muscle cells, neurons and β-pancreatic cells	Peroral administration	Yet to be clarified	Diazoxide decreases the release of insulin and is used to manage hypoglycemia caused by pancreas cancer, surgery, or other conditions
NN414 (Tifenazoxide) (Mannhold, 2004)	SUR1-selective KCO	Neurons and β-pancreatic cells	Peroral administration	Whether NN414 can cross BBB is uncertain. Given its lipophilic nature and small molecular weight (291.78 Da) a direct action of NN414 on central neurons is possible	Not approved for clinical use

^a^ Cromakalim, levcromakalim, pinacidil and diazoxide target mitochondrial K_ATP_, channel. Opening of mitochondrial K_ATP_, channels could improve mitochondrial ATP, production and lower Ca2+ overload.

^b^ KCOs, are lipophilic and small molecules (molecular weight <500 g/mol) that have several routes of administration. KCOs, are rapidly absorbed following oral administration with the time to peak plasma concentration being 0.5–1 h.

**TABLE 2:** Central intrathecal administration.

Author	Study design	Measurements	Interventions	Findings
Xia et al. (2014)	*In vivo* investigation using female Wistar rats	Mechanical stimulus-induced nociception: Mechanical withdrawal threshold (MWT) measurements performed using von Frey filaments on right hind paw Measurements 1, 3, 7, 9, 11, 13 and 15 days after surgery	Pinacidil (10–100 µg) or vehicle, 10 µL i.t.	The MWT was significantly lower in the bone cancer pain group than in the sham group after 3–12 days. No significant difference in MWT was observed between the sham and naïve control groups The vehicle had no significant effect on the MWT in the sham control and bone cancer pain groups Pinacidil did not exert a significant effect on MWT in the sham group Pinacidil significantly increased the MWT in the bone cancer pain group in a dose-dependent manner, which persisted for 30–60 min post-injection
	Bone cancer pain model: 10 µL Walker 256 cells (4 × 10^9^/L) injected in the tibia			
	Sham group: 10 µL D-Hanks injected			
	Naïve control group: Untreated			
	Intrathecal (i.t.) injection of pinacidil 15 days post-surgery			
Wu et al. (2011)	*In vivo* investigation using male Sprague-Dawley rats Peripheral nerve injury model: Chronic constriction injury (CCI) of the left sciatic nerve Sham operated group: Operated but no nerve injury Naïve control group: Untreated Pain induction test: Injections of cromakalim for 3 days starting 30 min pre-surgery Pain persistence test: Posttreatment with cromakalim as a single injection on day 10 Assessment of long-term effects following repetitive administration: posttreatment with cromakalim injections on days 10, 11, and 12 Diazoxide: a single injection on postoperative day 10	Heat- and mechanical stimulus-induced nociception: MWT measurements performed using von Frey filaments to each hind paw Measurement of withdrawal latency following application of thermal stimulus to each hind paw using an analgesiometer Baseline tests performed 2 days pre-surgery Assessment of drug effects upon induction of neuropathic pain: Postoperative days 1, 3, 4, and 5 Assessment of drug effects on persistence of neuropathic pain: Postoperative days 3, 7, and 10. Additional tests performed 1, 2, 4, 6, 24, 48, and 72 h after a single cromakalim injection on the day 10 Long-term effect of repetitive administration of cromakalim on persistent pain tests: Postoperative days 3, 7, and 10. Additional tests 2–4 h after each injection on postoperative days 10, 11, and 12, respectively, and continuing for 1, 2, and 3 days after termination of the last injection In naïve rats: Tests 1, 2, 4, 6, and 24 h after single cromakalim injection	Cromakalim (5, 10 or 20 µg) or vehicle, 20 µL i.t. Diazoxide (20 µg), or vehicle, 20 µL i.t.	CCI induced long-lasting thermal hyperalgesia and mechanical allodynia compared with sham operation Induction of neuropathic pain: Pretreatment with cromakalim (10 and 20 µg) delayed the onset of thermal hyperalgesia and mechanical allodynia in the CCI group for 1–2 days Persistence of neuropathic pain: Cromakalim (10 and 20 µg) suppressed thermal hyperalgesia and mechanical allodynia in the CCI group. A single injection of cromakalim on postoperative day 10 suppressed pain within 1 h, which then peaked at 6–8 h and returned within 24 h Long-term effect of repetitive administration: Cromakalim injections administered on postoperative days 10, 11, and 12, respectively, resulted in pain inhibition 4 h after each injection, with the pain returning within 2 days after the last injection Pretreatment or posttreatment with low-dose cromakalim (5 µg) failed to alter neuropathic pain sensitivity The vehicle failed to alter thresholds in CCI- or sham-operated groups Cromakalim did not alter thresholds in the naïve control group Diazoxide prevented thermal hyperalgesia in the CCI group for at least 2 h but not in the sham-operated group
Zhu et al. (2015)	*In vivo* investigation using male Sprague-Dawley rats: Postoperative pain model: Skin/muscle incision and retraction (SMIR) performed in the gracilis muscle of the right leg Sham-operated group: Same procedure but no retraction Naïve control group: Not operation Pinacidil injected i.t. 7 days post-surgery	Mechanical stimulus-induced nociception: MWT measurements performed using von Frey filaments to hind paws Measurements before and 1, 3, 5, 7, 10, 21, 28 and 32 days post-surgery	Pinacidil (4, 20 or 40 µg) or vehicle, 40 µL i.t.	There was no significant difference in basal MWT between the SMIR group, sham-operated group, or control group The MWT of the SMIR-operated group was decreased on day 1 and maintained for >21 days post-surgery. The SMIR-induced hypersensitivity in the ipsilateral paw was significant on postoperative day 3 and most prominent on day 10 when compared with baseline values No significant change in MWT in the sham or control groups, with no significant difference between the two groups Pinacidil markedly increased the MWT within 1 h and peaked at 2 h, with the pain returning within 3 h. Pinacidil 20 µg elicited optimal antinociceptive effects
Qian et al. (2023)	*In vivo* investigation performed with adult male C57BL/6 J mice Postoperative pain model: Plantar incision surgery performed on the plantaris muscle of the left hind paw Injections of cromakalim or vehicle (i.t.) were injected 30 min pre- and post-surgery every 24 h for 7 days Single injection of cromakalim (5 μg, 10 µL) or vehicle i.t. 30 min pre-surgery	Mechanical stimulus-induced nociception: MWT measurements were performed using von Frey filaments on the hind paws, with measurements at baseline and 6 h after each injection Single injection: Measurements every 2 h for 24 h total post-surgery	Cromakalim (1, 2.5 and 5 µg) or vehicle, 10 µL i.t.	Cromakalim (2.5 or 5 μg, 10 µL) significantly attenuated plantar incision-induced mechanical allodynia Compared with the vehicle, a single injection of cromakalim (5 μg, 10 µL) reduced nociception from approximately 2 h, peaking at 6–8 h, with the pain returning within 24 h
Koh et al. (2016)	*In vivo* investigation performed using male Sprague-Dawley rats Neuropathic pain model: Spinal nerve ligation (SNL) of left L5 and L6 spinal nerves distal to the dorsal root ganglion (DRG) Pre-nefopam group: Pretreated with nefopam (analgesic) (10, 30, and 60 mg/kg) or vehicle, 3 mL/kg intraperitoneal (i.p.) 20 min before SNL Post-nefopam group: Post-treated with nefopam (same doses) or vehicle, 3 mL/kg i.p. 7 days after SNL Pretreatment with pinacidil or vehicle i.t. 20 min before posttreatment with nefopam or vehicle Pinacidil was administered alone at the same doses	Mechanical stimulus- nociception: MWT measurements were performed using von Frey filaments to ipsilateral hind paw with nerve injury Measurements at baseline 1-day pre-surgery and 5, 9, 13, 17, 21, 25, and 29 days post-SNL in the pre-nefopam group Measurements at 30, 60, 90, and 120 min post-injection and 8, 9, 13, 17, 21, and 29 days post-SNL in the post-nefopam group	Pinacidil (10 and 30 µg) or vehicle, 10 µL i.t.	Nefopam reversed mechanical allodynia in SNL rats in a dose-dependent manner, as shown by elevated MWT, when compared with the control group. The effect was maintained until day 29 Compared with the vehicle treatment, pretreatment with high-dose nefopam (30 and 60 mg/kg) suppressed the development of mechanical allodynia in SNL rats, as shown by increased MWT Pinacidil at both doses enhanced the antiallodynic effect of nefopam by days 8 and 9 Pinacidil alone elicited a dose-dependent antiallodynic effect when compared with the vehicle
Cao et al., (2016b)	*In vivo* investigation performed using male mice (no breed listed) Morphine tolerance model: Morphine (opioid) (10 µg) or vehicle, 10 µL i.t. daily for 7 days Pretreatment with cromakalim (0.3, 1, or 3 µg) or vehicle i.t. 15 min before morphine administration Administration of cromakalim (3 µg) alone for 7 days	Heat stimulus-induced nociception: Measurements of tail-flick latency to thermal stimulus with hot water Measurements performed 30 min after every morphine injection	Cromakalim (0.3, 1 or 3 µg) or vehicle, 10 µL i.t.	There was no change in tail-flick latency or behavior when injected with cromakalim (3 µg) or the vehicle alone Mice repeatedly treated with morphine showed a time-dependent decrease in the maximum possible effect elicited by morphine Coadministration of cromakalim with morphine inhibited the reduced morphine analgesia in a dose-dependent manner, although the effect was only significant at 3 µg cromakalim
Song et al. (2011)	*In vivo* investigation performed using male Sprague-Dawley rats Neuropathic pain model: SNL of left L5 and L6 spinal nerves distal to the DRG Adenosine A1 receptor agonist N^6^-(R)-phenylisopropyladenosine (R-PIA) (0.5, 1, or 2 µg) or vehicle, 10 µL i.t. injected Diazoxide or vehicle-injected alone or 5 min before R-PIA injection	Mechanical stimulus-induced nociception: MWT measurements with von Frey filaments to hind paw ipsilateral to nerve injury. Measurements at baseline, 10, 20, 30, 40, 50, 60, 90 and 120 min after drug administration	Diazoxide (10, 30, and 100 µg) or vehicle, 10 µL i.t.	From 20 min onward, R-PIA elicited a dose-dependent antiallodynic effect compared with the vehicle. The effect of R-PIA (2 µg) was greater than that induced by lower doses from 30 min onward Diazoxide alone elicited a dose-dependent antiallodynic effect of similar magnitude to that of R-PIA (2 µg) Initially, high-dose R-PIA (2 µg) exerted a more profound effect than low-dose R-PIA (0.5 µg) with diazoxide (100 µg). Gradually, the effect of diazoxide increased until the animals receiving low-dose R-PIA and pretreated with diazoxide demonstrated a greater antiallodynic effect than high-dose R-PIA alone
Cheng et al. (2006)	*In vivo* investigation performed using male Wistar rats Postoperative pain model: Plantar incision surgery to the plantaris muscle in the right hind paw I.t. administration of 5 µL of drug, followed by 10 µL of saline Gabapentin (anticonvulsant) (100 µg) i.t. Adenosine triphosphate-sensitive potassium (KATP) channel openers, i.t.	Mechanical stimulus-induced nociception: MWT measurements performed using von Frey filaments (before, 2 h after incision, and 15, 30, 45, 60, 90, and 120 min after i.t. administration)	Pinacidil (100 µg or 300 µg) or vehicle, 5 µL i.t Diazoxide (600 µg or 1,200 µg) or vehicle, 5 µL i.t.	Gabapentin (100 µg) reduced allodynia in the postoperative pain model. The effect peaked after 45–60 min and lasted for more than 2 h Administration of pinacidil (100 or 300 µg), diazoxide (600 or 1,200 µg), and the vehicle did not alter allodynia in the postoperative pain model
Mixcoatl-Zecuatl et al. (2004)	*In vivo* investigation performed using female Wistar rats Neuropathic pain model: SNL of left L5 and L6 spinal nerves distal to the DRG Sham-operated group I.t. gabapentin (25–200 µg) or vehicle, 10 µL Positive control: i.t. pinacidil or vehicle MWT evaluation 15 min after i.t. injections	Mechanical induced nociception: MWT measurements performed using von Frey filaments Measurements every 30 min for 3 h	Pinacidil (1–30 µg) or vehicle, 10 µL i.t.	SNL-induced allodynia when compared with the sham-operated group Gabapentin or pinacidil, but not the vehicle, reduced tactile allodynia in a dose-dependent manner in the SNL group Gabapentin failed to reduce MWT in the sham-operated group The maximum antiallodynic effect was achieved with 10 µg pinacidil and 100 µg of gabapentin
Mixcoatl-Zecuatl et al. (2006)	*In vivo* investigation performed using female Wistar rats Neuropathic pain model: SNL of left L5 and L6 spinal nerves distal to the DRG Sham-operated group I.t. gabapentin (25–100 µg) or vehicle, 10 µL Positive control: i.t. K_ATP_ opener or vehicle MWT evaluation 15 min after i.t. injections	Mechanical induced nociception: MWT measurements performed using von Frey filaments Measurements every 30 min for 3 h	Diazoxide (3–100 µg) or vehicle, 10 µL i.t. Pinacidil (10 µg) or vehicle, 10 µL i.t.	Compared with the vehicle, i.t. administration of gabapentin (100 µg), diazoxide (100 µg), or pinacidil (10 µg) reduced SNL-induced tactile allodynia Gabapentin and diazoxide exerted dose-dependent effects

**TABLE 3:** Central intracerebroventricular administration.

Author	Study design	Measurements	Interventions	Findings
Narita et al. (1993)	*In vivo* investigation using male ddY mice Intracerebroventricular (i.c.v) administration of 5 µL morphine or U-50,488H Co-administration of cromakalim (0.2 µg) or vehicle with mu-opioid receptor agonist morphine or kappa-opioid receptor agonist U-50,488H. Median effective doses (ED_50_) reported in the article I.c.v administration of cromakalim alone	Heat stimulus-induced nociception: Measurements of paw-tap or paw-lick latency to thermal stimulus in the hot plate test Measurements before and 10 min post-injection	Cromakalim (0.2, 1, 5 or 10 µg) or vehicle, 5 µL i.c.v.	Cromakalim 10 µg elicited a significant antinociceptive effect when compared with vehicle Morphine provoked a potent antinociceptive effect, which was significantly increased by co-administration of 0.2 µg cromakalim. ED_50_ was 1.37 µg with vehicle and 0.48 µg with cromakalim Cromakalim did not significantly affect U-50,488H-induced antinociception
Kamei et al. (1994)	*In vivo* investigation using male ICR mice Diabetes group: model induction by administering intravenous (i.v.) streptozotocin (STZ) (200 mg/kg) Control group: injection of vehicle Pretreatment with insulin (5 U/kg) subcutaneous (s.c.) I.c.v. administration of cromakalim or vehicle alone or pretreatment with insulin	Heat stimulus-induced nociception: Measurements of tail-flick latency with heat lamp Measurements before and 30 min after cromakalim injection	Cromakalim (0.3, 1 and 3 µg) or vehicle, 5 µL i.c.v.	No significant difference in tail-flick latency between diabetic and non-diabetic mice Cromakalim (0.3 and 1 µg) dose-dependently increased tail-flick latency in non-diabetic mice when compared with the vehicle Cromakalim (even at the high dose of 3 µg) did not markedly impact tail-flick latencies in diabetic mice when compared with the vehicle No difference in cromakalim (3 µg)-induced antinociceptive effect between insulin-treated and untreated diabetic mice The cromakalim-induced antinociceptive effect was significantly lower in insulin-treated diabetic mice than in non-diabetic mice
Robles et al. (1996)	*In vivo* investigation using female Swiss CD1 mice Administration of 5-HT_1A_ agonists (5 mL/kg, s.c.) 30 min before the test: 8-OH-DPAT, lesopitron, buspirone and tandospirone Pretreatment with i.c.v. cromakalim or vehicle 30 min before test	Heat stimulus-induced nociception: Measurements of forepaw-licking latency to thermal stimulus using the hot plate. Measurements performed 30 min post-injections	Cromakalim (32 or 64 µg) or vehicle, 5 µL i.c.v.	In the hot plate test, 8-OH-DPAT (0.5–4 mg/kg), lesopitron (5–40 mg/kg), buspirone (10–80 mg/kg), and tandospirone (10–80 mg/kg) elicited antinociceptive effects in a dose-dependent manner Pretreatment with cromakalim resulted in the displacement of the dose-response curve of 8- OH-DPAT (0.125–2 mg/kg) to the left in a dose-dependent manner when compared with the vehicle Cromakalim potentiated the antinociceptive effect of lesopitron (10 mg/kg), buspirone (10 mg/kg), and tandospirone (20 mg/kg) in a dose-dependent manner
Ocaña et al. (1995)	*In vivo* investigation using female CD-1 mice Administration of mu-opioid receptor agonists (5 mL/kg, s.c.): morphine, buprenorphine, methadone, fentanyl, and levorphanol I.c.v. administration of cromakalim or vehicle immediately after opioid administration	Heat stimulus-induced nociception: Measurements of tail-flick latency to a thermal stimulus with a radiant heat source. Measurements performed at baseline, 10, 20, 30, 45, 60, 90 and 120 min post- injections	Cromakalim (4–64 µg) or vehicle, 5 µL i.c.v.	All mu-opioid receptor agonists induced a dose-dependent antinociceptive effect: buprenorphine (0.04–0.64 mg/kg), morphine (1–16 mg/kg), methadone (1–6 mg/kg), fentanyl (0.02–0.32 mg/kg), and levorphanol (0.2–3.2 mg/kg) Cromakalim i.c.v. enhanced the antinociceptive effect of morphine (1 mg/kg), methadone (2 mg/kg), and buprenorphine (0.04 mg/kg) Cromakalim did not significantly modify the antinociceptive effect of fentanyl (0.04 mg/kg) or levorphanol (0.4 mg/kg) A fixed dose of cromakalim (32 µg) displaced the dose-response curves of morphine, methadone, and buprenorphine to the left without increasing their maximum antinociceptive effect. This cromakalim dose did not significantly displace the dose-response curve of fentanyl or levorphanol Cromakalim did not significantly modify the tail-flick latency in control animals
Ocaña and Baeyens (1994)	*In vivo* with female CD-1 mice Administration of adenosine A1 receptor agonist R-PIA (0.5–8 mg/kg, s.c.) or vehicle, 5 mL/kg Cromakalim i.c.v. administered immediately after R-PIA injection	Heat stimulus-induced nociception: Measurements of tail-flick latency to thermal stimulus with tail-flick apparatus. Measurements performed at baseline and 10, 20, 30, 45, 60, 90, and 120 min post-injection	Cromakalim (16–64 µg) or vehicle, 5 µL i.c.v.	R-PIA produced an antinociceptive effect in a dose-dependent manner Cromakalim caused a dose-dependent increase in the antinociceptive effect of R-PIA Cromakalim (32 µg) displaced the R-PIA dose-response line to the left No dose of cromakalim caused a significant change in the tail-flick latency of control mice
Galeotti et al. (2001)	*In vivo* investigation using albino Swiss mice Administration of tricyclic antidepressants (10 mL/kg, s.c.) 30 min before the test: Clomipramine (25 mg/kg) and amitriptyline (15 mg/kg) I.c.v. injections of pinacidil or vehicle 15 min before the hot plate test	Heat stimulus-induced nociception: Measurements of paw licking latency in response to thermal stimulus on the hot plate Measurements before and 15, 30, 45, and 60 min after drug administration Hole-board test: Evaluation of activity and spontaneous ability Rota-rod test: Evaluation of motor function	Pinacidil (5 µg or 25 µg) or vehicle, 5 µL i.c.v.	Clomipramine and amitriptyline elicited an antinociceptive effect, increasing the pain thresholds in the hot plate test. The maximum analgesic effect occurred 30 min post-administration Pinacidil (25 µg) potentiated the antinociceptive effect of clomipramine and amitriptyline by increasing licking latency values when compared with the vehicle Low-dose pinacidil (5 µg) was ineffective Pinacidil (25 µg) administered alone had no analgesic effect Clomipramine and amitriptyline in doses used in the hot plate test did not alter motor function, as evidenced in the rota-rod test or upon assessing activity and spontaneous ability in the hole-board test High-dose clomipramine (45 mg/kg) and amitriptyline (30 mg/kg) affected the motor function Pinacidil (25 µg) modulated the pain threshold when administered with the tricyclic antidepressants without affecting the motor function, activity, and spontaneous ability when compared with the vehicle
Galeotti et al., (1999a)	*In vivo* investigation using Swiss albino mice Administration of vehicle or antihistamines: pyrilamine (5–15 mg/kg s.c.), diphenhydramine (10–20 mg/kg s.c.), and promethazine (3–6 mg/kg s.c.) Pretreatment with pinacidil or vehicle 15 min before the test or administered alone	Heat stimulus-induced nociception: Measurements of paw-licking latency to thermal stimulus with hot plate test. Reaction times were measured before, 15, 30, 45, and 60 min after the antihistamine injection Motor coordination evaluation using the rota-rod test Reaction times measured before, 15, 30, 45, and 60 min after the antihistamine injection	Pinacidil (25 µg) or vehicle, 5 µL i.c.v.	In the host-placed test, pyrilamine, diphenhydramine, and promethazine elicited a dose-dependent antinociceptive effect compared with the vehicle Pretreatment with pinacidil administered i.c.v. 15 min prior to testing potentiated the antinociceptive effect of the antihistamines by increasing the pain threshold when compared with the vehicle Pinacidil administered alone did not affect the licking latency when compared with the vehicle Pyrilamine, diphenhydramine, and promethazine did not affect the motor function of mice when compared with vehicle-treated mice. Treatment with high-dose pyrilamine (25 mg/kg s.c.), diphenhydramine (30 mg/kg s.c.), and promethazine (10 mg/kg s.c.) significantly affected motor function in the rota-rod test
Nakao et al. (1996)	*In vivo* investigation using male ddY mice I.c.v. administration of cromakalim or vehicle Stress-induced analgesia performed 10 min later Foot shock for 15 min Forced swimming for 3 min Psychological (communication box) for 5 min	Mechanical stimulus-induced nociception: Nociception measured using the tail-pinch method every 5 min for 15 min	Cromakalim (0,1–10 µg) or vehicle, 10 µL i.c.v.	Neither cromakalim nor vehicle exerted a significant effect on stress-induced analgesia
Ocaña et al. (1996)	*In vivo* investigation using female CD-1 mice S.c. injections (5 mL/kg) of: mu-opioid receptor agonist: Morphine (0.5–16 mg/kg) Alpha-2-adrenoceptor agonist: Clonidine (0.12–2 mg/kg) Gamma-aminobutyric acid(B) receptor agonist: Baclofen (2–16 mg/kg) Kappa opioid receptor agonist: U50,488H (2–16 mg/kg) Immediately followed by i.c.v. injections of cromakalim or vehicle	Heat stimulus-induced nociception Measurements of tail-flick latency to a thermal stimulus with a noxious beam of light Measurements at baseline and 10, 20, 30, 45, 60, 90 and 120 min after injections	Cromakalim (8–64 µg) or vehicle, 5 µL i.c.v.	Clonidine elicited a dose-dependent antinociceptive effect Cromakalim (32 µg) enhanced the clonidine-induced antinociceptive effect when compared with the vehicle, shifting the dose-response curve to the left Several cromakalim doses (8–64 µg) associated with a fixed dose of clonidine (0.25 mg/kg) enhanced the clonidine-induced antinociceptive effect in a dose-dependent manner when compared with the vehicle Cromakalim alone did not significantly modify the tail-flick latency compared with the vehicle Morphine elicited a dose-dependent antinociceptive effect Cromakalim (32 µg) enhanced the morphine-induced antinociceptive effect when compared with the vehicle, shifting the dose-response curve to the left Several cromakalim doses (8–64 µg) associated with a fixed dose of morphine (1 mg/kg) increased the morphine-induced antinociceptive effect in a dose-dependent manner when compared with the vehicle Both baclofen and U50,488H elicited a dose-dependent antinociceptive effect Cromakalim (32 µg) did not shift the dose-response curve and did not modify the antinociceptive effect of baclofen pr U50,488H when compared with the vehicle Several doses of cromakalim (16–64 µg) associated with fixed doses of baclofen (4 mg/kg) or U50,488H (2 mg/kg) did not modify the antinociceptive effect of these drugs compared to vehicle
Sood et al. (2000)	*In vivo* investigation using Swiss albino mice of either sex Diabetes groups: Intraperitoneal (i.p.) STZ-induced diabetes (200 mg/kg) Group I-IV: Vehicle treated s.c. Group V-VIII: Morphine treatment (4 and 8 mg/kg; s.c.) Group IX-XIV: Cromakalim treatment (0.3, 1, and 2 μg; i.c.v.) Group XV-XVI: Morphine treatment (4 mg/kg; s.c.) and cromakalim treated (0.3 µg; i.c.v.) Group XVII-XVIII: The diabetic group was treated with zinc insulin suspension (1 U/kg every 8 h for 3 days before treatment with morphine (4 mg/kg; s.c.) and cromakalim (1 μg; i.c.v.) Group XIX-XXII: Underwent surgical splenectomy and, after 48 h, treated with morphine (4 mg/kg; s.c.) and cromakalim (1 μg; i.c.v.)	Heat stimulus-induced nociception: Measurements of tail-flick latency to thermal stimulus with radiant heat lamp Measurements 0, 5, 15, 30, 45, 60, 90, and 120 min after drug administration	Cromakalim (0.3, 1, and 2 µg) or vehicle, 10 µL i.c.v.	Morphine increased the tail-flick latency time in both diabetic and non-diabetic mice when compared with the vehicle The diabetic group exhibited a significant decrease in morphine-induced antinociception when compared with non-diabetic mice Cromakalim increased the tail-flick latency in non-diabetic mice when compared with the vehicle Cromakalim enhanced the tail-flick latency in diabetic mice when compared with the vehicle, although a significant effect was only observed with high-dose treatment (2 µg) and was less effective than in non-diabetic mice Co-administration of cromakalim (0.3 µg; i.c.v.) and morphine (4 mg/kg) slightly increased the tail-flick latency time in non-diabetic mice, with no increase in diabetic mice Insulin treatment enhanced the antinociceptive effect of morphine and cromakalim in diabetic mice. The increase in tail-flick latency time was lower in insulin-treated diabetic mice than in non-diabetic mice The antinociceptive effect of cromakalim and morphine was increased in splenectomy-operated diabetic mice and was comparable with that of non-diabetic mice. The increased tail-flick latency in the splenectomy group was higher than that in the insulin-treated group and statistically significant

**TABLE 4:** Intraperitoneal administration.

Author	Study design	Measurements	Interventions	Findings
Christensen et al. (2020)	*In vivo* investigation using male C57Bl/6 J mice Intraperitoneal (i.p.) administration of levcromakalim or vehicle every other day (5 times in total)	Mechanical and heat stimulus-induced nociception: MWT measurements performed using von Frey filaments to left hind paw Measurements at baseline and every hour (h) for 4 h post-injection on days 1, 3, 5, 7, and 9	Levcromakalim (0.1, 0.5, and 1 mg/kg) or vehicle (10 mL/kg; i.p.)	Compared with the vehicle, levcromakalim induced hyperalgesia, decreasing the MWT in a dose-dependent manner. The effect was notable after the third dose, administered on day 5. The effect was most pronounced 2 h post-injection. The basal response to levcromakalim was significantly reduced on day 9 in the two highest dose groups but was reduced from day 5
Qian et al. (2016)	*In vivo* investigation using male Sprague-Dawley rats Postoperative pain model: SMIR operation in the gracilis muscle of the right leg Sham-operated group: Same procedure but no retraction Control group: No treatment Pinacidil injected i.p. 30 min prior to SMIR surgery	Mechanical stimulus-induced nociception: MWT measurements performed using von Frey filaments Measurements on day 1, 3, 7, and 12 post-SMIR	Pinacidil (25 μg/kg; i.p.)	SMIR induced mechanical allodynia on day 3, 7, and 12. No significant alterations on the first day compared to control group Pretreatment with pinacidil reversed the allodynia observed following SMIR. No significant reduction in MWT in pinacidil group compared to control group
Cao et al. (2015)	*In vivo* investigation using male Sprague-Dawley rats Postoperative pain model: SMIR operation in the gracilis muscle of the right leg Sham-operated group: Same procedure but no retraction Control group: No treatment Pinacidil administered at different doses pre-surgery	Mechanical induced nociception: MWT measurements with von Frey filaments. Measurements before and 1, 3, 7 and 12 days after surgery	Pinacidil (10, 25 and 50 μg/kg) i.p.	SMIR induced mechanical allodynia in a time-dependent manner when compared with the control No significant differences in MWT in the sham group pre- and post-surgery Pretreatment with pinacidil attenuated the SMIR-induced allodynia in a dose-dependent manner The was a significant reduction in MWT in the low-dose pinacidil group 3, 7, and 12 days post-surgery MWT was significantly reduced in the medium-dose pinacidil group 12 days post-surgery High-dose pinacidil completely blocked the SMIR-induced reduction in MWT at measurement time points
Vale et al. (2007)	*In vivo* investigation using male Swiss mice Mice injected with phosphodiesterase 5 inhibitor sildenafil (1–10 mg/kg; i.p.) or vehicle. 20 min later, zymosan injections (i.p.) Pretreatment: glibenclamide (K_ATP_ channel blocker; 0.1–1 mg/kg) peroral (p.o.) or vehicle 45 min before sildenafil Diazoxide (1 mg/kg; i.p.) or vehicle plus sildenafil Diazoxide (1 mg/kg; i.p.) or vehicle before glibenclamide	Zymosan-induced nociception: Writhing model: zymosan injected i.p. (1 mg, 0.2 mL) and nociception intensity evaluated by number of writhes 0–20 min post-injections	Diazoxide (1 mg/kg) or vehicle i.p.	Zymosan administration produced a writhing response Sildenafil inhibited the zymosan-induced writhing response in a dose-dependent manner, with 10 mg/kg eliciting the maximal effect Compared with the vehicle, pretreatment with glibenclamide reversed the antinociceptive effect of sildenafil in a dose-dependent manner Pretreatment with diazoxide before glibenclamide (1 mg/kg) antagonized the effect of glibenclamide on the antinociceptive effect of sildenafil Pretreatment with diazoxide enhanced the antinociceptive effect of sildenafil

**TABLE 5:** Intramuscular and epidural administration.

Author	Study design	Measurements	Interventions	Findings
Asano et al. (2000)	*In vivo* investigation using male Sprague-Dawley rats 50 µL epidural injections: morphine (1, 10, and 100 µg) and levcromakalim (10 and 100 µg) Other experiments with intramuscular (i.m.) levcromakalim after epidural administration of morphine	Heat stimulus induced nociception: Measurements of tail-flick latency to thermal stimulus with custom-made tail-flick apparatus Measurements at baseline and 5, 10, 20, 30, and 60 min after the last injection	Levcromakalim (10 and 100 μg, 50 μL; epidural) Levcromakalim (100 μg, 200 μL; i.m.)	Epidural administration of levcromakalim alone did not significantly increase tail-flick latency Epidural low-dose morphine (1 µg) did not induce an apparent antinociceptive effect. High-dose epidural levcromakalim (100 µg) produced a significant potentiation of the effect of epidural morphine and caused a left shift in the dose-response curve Levcromakalim (100 µg) did not potentiate the antinociceptive effect of epidural morphine (1 µg) when administered i.m. Epidural morphine increased the tail-flick latency in a dose-dependent manner
Niu et al. (2011)	*In vivo* investigation using male and female Wistar rats Pretreatment of masseter muscle with pinacidil (2, 20, 100, and 300 µg) 10 µL i.m. or vehicle After 5 min, 100 µL of TRPV1 receptor agonist capsaicin (0.1%) was administered Diazoxide (100 and 300 µg) 10 µL i.m. was examined in the same manner High-dose pinacidil (300 µg) injected into the masseter muscle contralaterally in capsaicin-injected muscle in a separate group Female rats pretreated with medium-dose pinacidil (20 µg) administered during the proestrus or diestrus phase	Mechanical induced nociception: Measurements of thresholds using von Frey aesthesiometer until ipsilateral hind paw shaking when force applied to masseter muscle. Measurements of baseline and 15, 30, 45, 60, and 90 min after drug treatment	Pinacidil (2, 20, 100, and 300 μg; i.m.) Diazoxide (100 and 300 μg; i.m.)	No difference between sexes in baseline thresholds Both sexes developed acute mechanical hypersensitivity in the masseter muscle after capsaicin injections, although female rats showed higher hypersensitivity Pretreatment with pinacidil attenuated the capsaicin-induced hypersensitivity in a dose-dependent manner in male rats. Pinacidil (20 µg) blocked the hypersensitivity reaction completely. The same dose was ineffective in female rats Pinacidil (300 µg) significantly attenuated capsaicin-induced hypersensitivity in female rats Pinacidil (300 µg) had no effect when injected contralaterally Pinacidil (20 µg) did not attenuate capsaicin-induced hypersensitivity in female rats during the proestrus or diestrus phases of the estrus cycle Pretreatment with diazoxide attenuated capsaicin-induced hypersensitivity in a dose-dependent manner in male rats Diazoxide (300 µg) failed to attenuate capsaicin-induced hypersensitivity in female rats
Cairns et al. (2008)	*In vivo* investigation using Sprague-Dawley rats Two injections (10 µL) within 30 min intervals in masseter muscle I.m. injections of potassium chloride (KCl; 2.0 M), followed by either KCl alone or KCl + pinacidil (1 or 0.1 mg/mL) I.m. injections of hypertonic saline (HS), followed by HS + pinacidil (0.1 mg/mL)	Trigeminal primary afferent fiber activity (masseter muscle) under surgical anesthesia measured using microelectrodes (baseline and monitored for 10 min after each injection) Mechanical threshold measurements performed using von Frey filaments applied to masseter muscle (baseline, 1 min post-injection and for 10 min with 1-min intervals)	Pinacidil (0.1 mg/mL), 10 µL alone or as second injection (1 or 0.1 mg/mL) i.m.	Repeated injection of HS or KCl caused afferent discharge Co-injection of pinacidil (1 mg/mL) with KCl suppressed the KCl-induced afferent discharge, but this dose also made the afferents unresponsive to mechanical stimulation of the muscle after 10–20 min KCl alone did not impact the responsiveness of afferents to mechanical stimulation of the muscle Co-injection of pinacidil (0.1 mg/mL) with KCl increased the KCl-induced afferent discharge, and afferents were responsive to mechanical stimulation Pinacidil alone elevated the mechanical threshold by 20% up to 10 min post-injection Pinacidil (0.1 mg/mL) did not significantly impact the HS-induced afferent discharge

**TABLE 6:** Intraplantar administration.

Author	Study design	Measurements	Interventions	Findings
Du et al. (2011)	*In vivo* investigation using male Sprague-Dawley rats Group administered intraplantar (i.pl.) injections of adenosine triphosphate sensitive potassium (K_ATP_) channel openers or vehicle 5 min before i.pl. injections of bradykinin (BK) or vehicle Group administered K_ATP_ channel openers or vehicle 8 min before withdrawal measurements Control experiments: Injection of K_ATP_ channel opener to contralateral paw of BK injections Phentolamine (vasodilator) injected 5 min before BK injection	BK, heat, and mechanical stimulus induced nociception: Rats injected with BK (200 µM) or vehicle (50 μL; i.pl). Nocifensive behavior recorded for 30 min Measurements of withdrawal latency of the hind paw following thermal stimulus with heat lamp Mechanical withdrawal threshold (MWT) measurements performed using von Frey filaments applied to the hind paw Measurements performed 8 min post-injections	Pinacidil (10 µM) or vehicle (50 µL i.pl.) Diazoxide (100 µM) or vehicle (50 µL i.pl.)	Compared with the vehicle, BK i.pl. resulted in strong nocifensive behavior Co-administration of diazoxide or pinacidil with BK reduced the BK-induced nocifensive behavior by 50%. Vehicle treatment had no effect on BK-induced nocifensive behavior Control experiments: Pinacidil injected into contralateral paw did not affect the BK-induced nocifensive behavior. Phentolamine did not affect the BK-induced nocifensive behavior Diazoxide and pinacidil significantly increased the withdrawal latencies upon applying thermal stimulus when compared with the vehicle Diazoxide and pinacidil significantly increased the MWT when compared with the vehicle
Alves et al. (2004a)	*In vivo* investigation using male Wistar rats Rats injected subcutaneous (s.c.) with prostaglandin E_2_ (PGE_2_) or vehicle (0.1, 0.5, and 2 µg) to plantar surface of right hind paw Diclofenac (non-steroid anti-inflammatory drug) (25, 50, 100, and 200 µg) or vehicle (100 µL) injected to right hind paw 2 h) after PGE_2_ NG-Nitro L-arginine (nitrogen oxide synthase inhibitor) (50 μg; 100 µL) i.pl. injected to right hind paw 1 h before diclofenac Diazoxide administered 45 min after diclofenac to the right hind paw Control: PGE_2_ injected into both hind paws. After 2 h, diclofenac was administered into the left or right hind paw	Mechanical stimulus induced nociception: Paw pressure measured using an analgesy-meter applied to right hind paw. Measurements of nociceptive thresholds on right hind paw before and 3 h after injection of PGE_2_	Diazoxide (300 μg; 100 µL i.pl.)	PGE_2_ reduced the nociceptive threshold to mechanical stimuli in a dose-dependent manner when compared with the vehicle Diclofenac injected into the right hind paw elicited a dose-dependent antinociceptive response to PGE_2_ (2 µg)-induced hyperalgesia. No statistical difference was detected between the 200 and 100 µg doses in terms of counteracting PGE_2_-induced hyperalgesia Diclofenac 100 µg injected into the contralateral paw did not elicit an antinociceptive effect in the right paw. Diclofenac injected into the contralateral paw at 400 µg evoked an antinociceptive effect in the right paw NG-Nitro L-arginine antagonized the antinociceptive effect of diclofenac (100 µg) Diazoxide reversed the antagonistic effect of NG-Nitro L-arginine
Fisher et al. (2019)	*In vivo* investigation using adult C57B16 mice Neuropathic pain model: SNL of left L5 Right L5 spinal nerve left intact as control Morphine (opioid) tolerance test: morphine (15 mg/kg) 100 μL s.c., twice daily for 5 days I.pl. injections of K_ATP_ opener or vehicle 10 min after morphine injections	Mechanical induced nociception: MWT measurements performed on the ipsilateral hind paw using von Frey filaments Baseline measurements and every day 30 min after morphine administration. On day 6, 18 h after last morphine injection and 20 min after the last K_ATP_ opener or vehicle injection	Diazoxide (100 µM solution) or vehicle (10 μL; i.pl.) NN414 (100 µM solution) or vehicle (10 μL; i.pl.) Pinacidil (100 µM) or vehicle (10 μL; i.pl.)	NN414 and diazoxide i.pl. attenuated morphine tolerance when compared with the vehicle Pinacidil i.pl. did not significantly attenuate morphine tolerance when compared with the vehicle On day 6, NN414 administration significantly enhanced MWT in morphine-withdrawn mice when compared with vehicle administration. Pinacidil did not exert a significant effect on the MWT when compared with the vehicle
Ghorbanzadeh et al. (2019)	*In vivo* investigation using male Wistar rats Formalin (2.5%) 50 µL subplantar injection to the right hind paw Pretreatment with carbamazepine (anticonvulsant; 100, 300, and 1,000 µg) or vehicle (50 µL) to the ipsilateral paw 20 min before formalin administration Pretreatment with diazoxide 10 min before carbamazepine administration Control experiment with carbamazepine (1,000 µg) 50 µL to contralateral paw 20 min before formalin injection into right hind paw	Formalin-induced nociceptive behavior: Number of flinches per min, measured every 5 min for 60 min. The first 5 min considered neurogenic phase, while 15–60 min considered inflammatory phase	Diazoxide (400 μg; 50 μL, i.pl.)	Formalin caused a classic pattern of flinching behavior. A biphasic time course with an early phase (neurogenic phase) within 5 min post-injection and a late phase (inflammatory phase) from 15 min to 1 h post-injection Administration of carbamazepine to the right hind paw reduced the formalin-induced nociceptive behavior in a dose-dependent manner during both the early and late phases of the test. At 100 μg, carbamazepine elicited a subeffective dose, with 300 µg determined as an effective dose. Carbamazepine injected contralaterally did not impact the formalin-induced nociceptive behavior Combination of diazoxide and the subeffective dose of carbamazepine elicited an antinociceptive effect in the early and late phases of formalin test
Alves et al. (2004b)	*In vivo* investigation using male Wistar rats S.c. injection of PGE_2_ (0.1, 0.5, and 2 μg; 100 µL) administered to the plantar surface of right hind paw Vasodilator sodium nitroprusside (125 μg; 100 µL) or cGMP analog dibutyryl-cGMP (db-cGMP; 50 μg, 100 µL) administered s.c. to the right hind paw 2 h after PGE_2_ injection Diazoxide administered s.c. to the right hind paw 2 h 45 min after PGE_2_ injection or to the contralateral paw	Mechanical stimulus-induced nociception Paw pressure test with analgesy meter applied to the right hind paw before and 3 h after PGE_2_ injection. Measurements of changes in the nociceptive threshold	Diazoxide (20, 38, 75, 150, 300 and 600 µg) 100 µL i.pl.	PGE_2_ i.pl. dose-dependently reduced nociceptive withdrawal threshold when compared with the control threshold, with a peak of effect observed 3 h after administration Diazoxide dose-dependently reduced the effect of PGE_2_ (2 µg) Diazoxide (300 µg) did not induce an antinociceptive effect in the right paw when injected into the contralateral paw Both sodium nitroprusside (125 µg) and diazoxide (20 µg) evoked an antinociceptive effect, respectively, on PGE_2_-induced hyperalgesia. Co-administration at the same doses elicited a marked inhibitory effect against PGE_2_-induced hyperalgesia Both db-cGMP (50 µg) and diazoxide (20 µg) produced an antinociceptive effect, respectively, against PGE_2_-induced hyperalgesia. Co-administration of db-cGMP and diazoxide elicited a marked inhibitory effect against PGE_2_-induced hyperalgesia
Picolo et al. (2003)	*In vivo* investigation using male Wistar rats I.pl. administration of PGE_2_ (100 ng) or λ-carrageenan (200 µg) to the hind paw After 2 h, peroral (p.o.) administration of *Crotalus durissus terrificus* snake venom (200 μg/kg) Control (confirmation of local antinociceptive action): K_ATP_ openers i.pl.	Mechanical stimulus-induced nociception Paw pressure test performed using pressure apparatus applied to the right hind paw Pain withdrawal thresholds before and 3 h after PGE_2_ or λ-carrageenan administration	Diazoxide (50–200 μg; i.pl.) or vehicle Pinacidil (50–200 μg, i.pl.) or vehicle	I.pl. administration of PGE_2_ or λ-carrageenan -induced hyperalgesia and reduced pain thresholds Treatment with snake venom-induced antinociception and enhanced pain thresholds Both diazoxide and pinacidil elicited a long-lasting increase in pain thresholds in non-treated rats and antinociception in rats treated with λ-carrageenan or PGE_2_ in a dose-dependent manner
Ghorbanzadeh et al. (2018)	*In vivo* investigation using male Wistar rats S.c. administration of formalin (50 µg of 2.5% solution in saline) to the plantar surface of the right hind paw Pretreatment with 50 µL fluoxetine (selective serotonin reuptake inhibitor) (10, 30, 100, and 300 µg) or vehicle to right hind paw 20 min before formalin administration Pretreatment with diazoxide or vehicle 10 min before fluoxetine administration Control experiment with maximum fluoxetine dose administered to the contralateral hind paw	Formalin-induced nocifensive behavior: Number of flinches per min, measured every 5 min for 60 min total	Diazoxide (100, 200, and 400 μg; i.pl.) or vehicle	Formalin elicited classic flinching behavior: A biphasic time course, with an early phase (neurogenic phase) within 5 min post-injection and a late phase (inflammatory phase) from 15 min to 1 h after injection Fluoxetine reduced flinching behavior ipsilaterally in a dose-dependent manner in both phases. No effect was observed in the contralateral paw High-dose diazoxide potentiated the antinociceptive effect of fluoxetine (100 µg) in the late phase of the formalin test but not in the early phase
Sachs et al. (2004)	*In vivo* investigation using male Wistar rats Acute hypernociception model: I.pl. administration of PGE_2_ (100 ng) or vehicle. Diazoxide i.pl. was administered 2 h after PGE_2_ administration Persistent hypernociception model (chronic inflammatory pain model): I.pl. administration of PGE_2_ (100 ng) or vehicle daily for 14 days. Diazoxide i.pl. was administered 5 days after discontinuing PGE_2_	Paw pressure test with constant pressure applied to the right hind paw until a “freezing reaction” Acute hypernociception measured before and 3 h after PGE_2_ administration Persistent nociception measured for 30 days	Diazoxide (600 µg) or. vehicle (i.pl.)	In the acute hypernociception model, treatment with diazoxide induced an antinociceptive effect when compared with the control Diazoxide blocked persistent hypernociception, resulting in the antinociceptive quiescent phase of persistent hypernociception (where a small nociceptive stimulus could restore the intensity of hypernociception)
Ortiz et al. (2002)	*In vivo* investigation using female Wistar rats S.c. administration of formalin (50 µL of 1% solution in saline) to the dorsal side of the right hind paw Pretreatment with pinacidil 20 min before formalin administration	Formalin-induced nociceptive behavior: Number of flinches per min, measured every 5 min for 60 min total	Pinacidil (1–50 µg) s.c. to dorsal side of right hind paw	Formalin produced a typical biphasic pattern of flinching behavior: First phase within 10 min, and second phase after 15 min to 1 h Pinacidil elicited a dose-dependent antinociceptive effect against formalin-induced pain during the second phase of the formalin test
Du et al. (2014)	*In vivo* investigation using male Sprague-Dawley rats Pinacidil or vehicle applied to the dorsal root ganglion (DRG) of L5 Immediately after administering BK (200 µM) or vehicle (50 μL, i.pl.) to the ipsilateral hind paw	BK-induced nociception: Nocifensive behavior - flinching, licking, and biting the injected paw was analyzed for 30 min	Pinacidil (200 µM) or vehicle (5 µL) in DRG	I.pl. administration of BK induced strong nocifensive behavior, with no such effects observed following pretreatment with the vehicle Pretreatment with pinacidil significantly attenuated the BK-induced nocifensive behavior when compared with the vehicle

**TABLE 7:** Central intrathecal and central intracerebroventricular administration.

Author	Study design	Measurements	Interventions	Findings
Zushida et al. (2002)	*In vivo* investigation using male ICR-mice STZ-induced diabetes (200 mg/kg; i.v.) Non-diabetic control group: Vehicle injection After 2 weeks: Pinacidil (i.c.v or i.t.)	Heat-induced nociception Measurements of tail-flick latency to a thermal stimulus with a beam of light. Measurements 10 min (min) after injection	Pinacidil (3–30 µg) i.c.v. Pinacidil (10 or 30 µg) i.t.	Diabetic mice had lower tail-flick latencies than non-diabetic mice Pinacidil i.c.v. dose-dependently increased the tail-flick latencies in both diabetic and non-diabetic mice. No significant differences in the antinociceptive effect of pinacidil in the two groups Pinacidil (10 or 30 μg; i.t.) dose-dependently increased the tail-flick latencies in the diabetic group but did not produce a significant antinociceptive effect in the non-diabetic group. Pinacidil (100 μg; i.t.) induced a significant antinociceptive effect in non-diabetic mice, although barely notable Treatment with pinacidil elicited a significant antinociceptive effect in diabetic mice when compared with that in non-diabetic mice

**TABLE 8:** Central intracerebroventricular, intraperitoneal, intraplantar, and peroral administration.

Author	Study design	Measurements	Interventions	Findings
Christensen et al. (2022)	*In vivo* investigation using male and female C57Bl/6 J wild-type (WT) mice I.p. injections of levcromakalim every other day, administered 2 or 6 times I.c.v. injections of levcromakalim or vehicle repeated twice, separated by a 1-day interval I.pl. injections of levcromakalim or vehicle between foot pads of the right hind paw, repeated twice, separated by a 1-day interval. HS is used as a positive control. No injection to the contralateral paw for control purposes	Heat- and mechanical stimulus-induced nociception Levcromakalim i.p. or i.c.v.: MWT measurements performed using von Frey filaments on hind paws. Measurements 120 min post-injection on days 1 and 3 Hind paw withdrawal- and licking latency time performed using the hot plate test. Measurements performed 20 and 135 min post-injection on days 1 and 3 Levcromakalim i.pl.: MWT measurements performed using von Frey filaments applied to the hind paws 20 and 120 min post-injection on days 1 and 3	Levcromakalim (1 mg/kg), 10 mL/kg i.p Levcromakalim (10 µg) or vehicle, 5 µL i.c.v Levcromakalim (2.5 µg) or vehicle (20 µL i.pl.)	In the hot plate test, i.p. administration of levcromakalim induced no response after 20 min. MWT was significantly lower 120 min after the second injection. Heat hypersensitivity developed after the second injection on day 3, with decreasing withdrawal latency time In the hot plate test, i.c.v. administration of levcromakalim increased withdrawal latencies after 20 min. No significant effect was observed after the second injection on day 3. There was no difference in MWT when compared with the vehicle No response was observed following intraplantar levcromakalim administration into the right hind paw. No response was observed in the contralateral paw. HS-induced hypersensitivity in the ipsilateral paw
Galeotti et al. (1999b)	*In vivo* investigation using male Swiss albino mice 10 mL/kg subcutaneous (s.c.) injections of alpha-2-adrenoceptor agonists: Clonidine (0.05–0.50 mg/kg) Guanabenz (0.05–1.0 mg/kg) Control group: Vehicle injection Pretreatment with K_ATP_ openers 15 min before s.c. injections	Heat stimulus-induced nociception: Measurements of the hind paw-licking latency to thermal stimulus in the hot plate test. Measurements at baseline, 15, 30, 45, and 50 min after clonidine treatment or 15, 30, 45, 60, 75, 90, 120, and 180 min after guanabenz treatment Motor coordination evaluation determined using the rota-rod test, performed simultaneously with the hot plate test	Pinacidil (25 μg, 5 μL; i.c.v.) Diazoxide (100 mg/kg) 10 mL/kg p.o. (esophageal injection)	In the hot plate test, clonidine (0.08–0.20 mg/kg) and guanabenz (0.05–0.50 mg/kg) elicited a dose-dependent antinociceptive effect Pretreatment with pinacidil and diazoxide potentiated the antinociceptive effect of 0.125 mg/kg clonidine and 0.30 mg/kg guanabenz. At various doses of clonidine (0.05–0.20 mg/kg) and guanabenz (0.05–0.30 mg/kg), pinacidil shifted the dose-response curves to the left None of the KATP modulators modified the licking latency values when administered alone In the rota-rod test, high doses of clonidine (0.50 mg/kg) or guanabenz (1.0 mg/kg) increased the number of falls when compared with the control group. Clonidine (0.125 and 0.20 mg/kg) and guanabenz (0.30 and 0.50 mg/kg) did not impair the motor function of the mice when compared with the control group

**TABLE 9:** Central intrathecal, intraperitoneal, and intraplantar administration.

Author	Study design	Measurements	Interventions	Findings
Shen et al. (2015)	*In vivo* investigation using male Sprague-Dawley rats Postoperative pain model: SMIR operation in the gracilis muscle of the right leg Sham-operated group: Same procedure but no retraction Naïve control group: No operation Pinacidil injected intraperitoneal (i.p.) 30 min before SMIR or i.t. 7 days post-SMIR	Mechanical stimulus-induced nociception MWT measurements performed using von Frey filaments applied to the hind paws Following i.p. pinacidil administration, MWT was measured over the following month Following i.t. pinacidil administration, MWT was measured for 3 h	Pinacidil (4, 20, or 40 µg) or vehicle (20 μL; i.t.) Pinacidil (10, 25 or 50 μg/kg) or vehicle (i.p.)	The MWT of the SMIR-operated group was transiently decreased >21 days post-surgery. No change in MWT was observed in the sham-operated group I.p. administration of pinacidil prior to SMIR dose dependently suppressed the allodynic effect of SMIR (reduction in MWT) compared with the vehicle Seven days after SMIR, pinacidil i.t. reversed the allodynic effect of SMIR in a dose-dependent manner when compared with the vehicle
Luu et al. (2019)	*In vivo* investigation using adult C57Bl6 mice Neuropathic pain model: SNL of left L5 and L6 spinal nerves distal to the DRG Control group: Uninjured K_ATP_ openers or vehicle administered intraplantar (i.pl.) or i.t. after SNL	Mechanical- and thermal stimulus-induced nociception Measurements of flinching/withdrawal latency upon application of thermal stimulus using glass plate apparatus (radiant paw withdrawal assay) MWT measurements performed using von Frey filaments applied to the hind paws Measurements of thresholds and latencies at baseline, 3, 15, 30, 45, and 60 min after drug administration Mice mobility and activity assessed using the open field test. Measurements before (15 min) and after (30 min) injection	Diazoxide 10 µL i.pl. and 10 µL i.t. NN414 10 µL i.pl. and 10 µL i.t. Pinacidil 10 µL i.pl. and 10 µL i.t. Levcromakalim 10 µL i.pl and 10 µL i.t. The doses of K_ATP_ openers have not been reported	The i.pl. administration of diazoxide and NN414, respectively, enhanced the MWT in the SNL group compared with the vehicle-treated group. Thermal thresholds were not significantly increased I.pl. administration of pinacidil and levcromakalim, respectively, did not significantly alter mechanical or thermal withdrawal latencies when compared with the vehicle The un-injected contralateral paw did not demonstrate any significant changes in mechanical or thermal withdrawal thresholds The i.t. administration of diazoxide, NN414, and levcromakalim, respectively, significantly increased MWT in the SNL group compared with the vehicle-treated group Open field test: Compared with the pre-vehicle injection group, the vehicle-administered (i.pl.) SNL group exhibited decreased distance traveled, reduced average velocity, and increased immobile time, indicating that the animals were hypersensitive after i.pl. injection. Diazoxide (100 μM; i.pl.) attenuated the effect on distance traveled and average velocity

## 4 Discussion

The main findings of the present systematic review were that KCOs can attenuate induced pain in various animal models and potentiate the effects of analgesics. To present a clear overview of the results, we divided these findings based on the route of administration. Studies of KCOs administered to the central nervous system have been divided into central intrathecal (i.t.) and central intracerebroventricular (i.c.v.) administration.

### 4.1 Central i.t. administration

Pinacidil, diazoxide, cromakalim, levcromakalim, and NN414 have been tested against bone cancer pain (Xia et al., 2014), postoperative pain (Shen et al., 2015; Zhu et al., 2015; Qian et al., 2023; Cheng et al., 2006) neuropathic pain (Koh et al., 2016; Mixcoatl-Zecuatl et al., 2004; Mixcoatl-Zecuatl et al., 2006; Song et al., 2011; Luu et al., 2019) and peripheral nerve injury (Wu et al., 2011). In chronic pain models, KCO administration was found to attenuate pain sensation and increase nociceptive thresholds (Xia et al., 2014; Shen et al., 2015; Zhu et al., 2015; Qian et al., 2023; Cheng et al., 2006; Koh et al., 2016; Mixcoatl-Zecuatl et al., 2004; Mixcoatl-Zecuatl et al., 2006; Song et al., 2011; Luu et al., 2019). Pinacidil, diazoxide, and cromakalim did not modulate the baseline pain threshold in sham-operated or naïve animals (Wu et al., 2011; Xia et al., 2014). Induction of persistent pain decreased K_ATP_ channel activity and downregulated K_ATP_ channel expression in the spinal cord (Xia et al., 2014; Shen et al., 2015; Zhu et al., 2015). By acting on the remaining K_ATP_ channels, pinacidil attenuated the induced pain (Xia et al., 2014) and potentiated the analgesic effect of intraperitoneal (i.p.) administered nefopam (Mixcoatl-Zecuatl et al., 2006). Diazoxide reportedly potentiates the antiallodynic effects of N6-R-phenylisopropyladenosine (R-PIA) (Song et al., 2011). Interestingly, i.t. pinacidil administration elicited an antinociceptive effect in diabetic and non-diabetic mice (Zushida et al., 2002), while cromakalim was found to attenuate morphine tolerance (Cao et al., 2016a). These results indicate that the central activation of K_ATP_ channels exerts an antinociceptive effect. Conversely, Cheng et al. found that neither pinacidil nor diazoxide altered allodynia in a postoperative pain model (Cheng et al., 2006).

### 4.2 Central i.c.v. administration

Intracerebroventricular, administration of pinacidil potentiated the antinociceptive effect of several drugs administered subcutaneously (s.c.): tricyclic antidepressants (Galeotti et al., 2001), antihistamines (Galeotti et al., 1999a) and alpha-2-adrenoceptor agonists (Galeotti et al., 1999b). In the absence of any concurrently administered drugs, i.c.v. administration of pinacidil did not induce an antinociceptive effect.

Furthermore, i.c.v. administration of cromakalim potentiated the antinociceptive effect of s.c. administered mu-opioid receptor agonists morphine (Ocaña et al., 1995; Ocaña et al., 1996), buprenorphine, and methadone (Ocaña et al., 1996), but did not alter the antinociceptive effect of fentanyl or levorphanol (Ocaña et al., 1996). I.c.v. cromakalim also potentiated the subcutaneous effect of 5-HT_1A_ receptor agonists (Robles et al., 1996), R-PIA (Ocaña and Baeyens, 1994) and clonidine (Ocaña et al., 1996). However, cromakalim did not alter the effects of s.c. administered U50,488H (a selective human kappa opioid receptor agonist) (Narita et al., 1993) and baclofen (Ocaña et al., 1996). Furthermore, cromakalim potentiated the effects of i.c.v. morphine, but not i.c.v. U50,488H (Narita et al., 1993). Collectively, these results suggest that K_ATP_ channel opening plays a role in mediating the analgesia of various types of drugs, with K_ATP_ channels functioning as downstream targets of receptor activation by these drugs. Considering cromakalim-induced analgesia when administered alone in naïve or control animals, the results were ambiguous. Some reported that cromakalim could induce an analgesic effect (Narita et al., 1993; Kamei et al., 1994; Sood et al., 2000), whereas others detected no such effect (Ocaña et al., 1995; Ocaña et al., 1996; Ocaña and Baeyens, 1994).

Three studies investigated the analgesic effect of i.c.v. pinacidil (Zushida et al., 2002) and i.c.v. cromakalim (Kamei et al., 1994; Sood et al., 2000) in diabetes models. Sood et al. (2000) reported that cromakalim induced substantial antinociception in diabetic mice, although to a lesser extent than that in non-diabetic mice. Moreover, cromakalim potentiated morphine-induced antinociception in the non-diabetic group but not in the diabetic group (Sood et al., 2000). This finding is in line with previous studies reporting that cromakalim can elicit a substantial antinociceptive effect in non-diabetic mice but not in diabetic mice, even after insulin treatment (Kamei et al., 1994). One potential explanation for this difference is the reduced activity or expression of supraspinal K_ATP_ channels in diabetes. Hyperglycemia and spleen-derived factors have been suggested to play a role in the reduced K_ATP_ channel activity (Kamei et al., 1994; Sood et al., 2000). The application of pinacidil provoked an antinociceptive effect in both diabetic and non-diabetic mice, with no marked differences in the potency ratio between the two groups (Zushida et al., 2002). I.c.v. administration of levcromakalim elicited an acute antinociceptive effect on heat-induced nociception without altering mechanical withdrawal thresholds (Christensen et al., 2022).

### 4.3 I.p. and peroral administration

Intraperitoneal administration of pinacidil could attenuate mechanical allodynia in postoperative pain models (Shen et al., 2015; Qian et al., 2016; Cao et al., 2015). I.p. diazoxide enhanced the antinociceptive effect of sildenafil against zymosan-induced nociception (zymosan is known to induce inflammatory pain) (Vale et al., 2007). Peroral (p.o.) administration of diazoxide potentiated the antinociceptive effect of s.c. administered alpha-2-adrenoceptor agonists clonidine and guanabenz but did not produce a notable effect when administered alone (Galeotti et al., 1999b). I.p. administration of levcromakalim (Christensen et al., 2022; Christensen et al., 2020) induced both thermal and mechanical hyperalgesia. These results differ from those of other KCOs and are further discussed below in relation to human trials.

### 4.4 Intramuscular (i.m.) and epidural administration

Intramuscular administration of pinacidil or diazoxide in the masseter muscle attenuated capsaicin-induced hypersensitivity (Niu et al., 2011). Pinacidil injection into the masseter muscle was shown to increase mechanical thresholds in trigeminal afferents (Cairns et al., 2008). These results suggest that K_ATP_ channels participate in the pain transmission of trigeminal afferent fibers and that the opening of these channels attenuates orofacial muscle pain.

Epidural co-administration of levcromakalim and morphine (Asano et al., 2000) potentiated the effect of morphine, although levcromakalim alone did not exert substantial antinociceptive effects (Asano et al., 2000). The observed potentiation may be due to the activation of mu-opioid receptors at the spinal cord level. Given that dissolving a higher dose of levcromakalim for epidural injection was not feasible, and with the dura mater functioning as a barrier, levcromakalim may fail to reach the spinal cord tissue (Asano et al., 2000). Notably, i.m. administration of the same dose of levcromakalim did not potentiate the effect of epidural morphine, indicating that the potentiation of morphine analgesia occurs at the spinal cord level (Asano et al., 2000).

### 4.5 Intraplantar administration

Pinacidil and diazoxide were co-applied to the paws with pain inducers, such as bradykinin (Du et al., 2011), formalin (Ghorbanzadeh et al., 2019; Ghorbanzadeh et al., 2018; Ortiz et al., 2002), λ-carrageenan, and prostaglandin E2 (PGE_2_) (Alves et al., 2004a; Alves et al., 2004b; Picolo et al., 2003; Sachs et al., 2004). Pinacidil and diazoxide were found to exert antinociceptive effects on pain induced by these agents and on heat- and mechanical-induced nociception. In formalin tests, pinacidil and diazoxide only influenced the late phases of the tests (Ghorbanzadeh et al., 2018; Ortiz et al., 2002). Notably, diazoxide potentiated the antinociceptive effect of carbamazepine against formalin-induced pain, with effects observed in both early and late phases (Ghorbanzadeh et al., 2019). Pinacidil applied to the dorsal root ganglion of the 5th lumbar nerve (L5) attenuated bradykinin-induced nociceptive behavior (Du et al., 2014). These results suggest that K_ATP_ channels are involved in peripheral antinociception of primary afferent neurons.

The effect of pinacidil on morphine tolerance was examined in a neuropathic pain model, revealing that pinacidil did not substantially impact morphine tolerance (Fisher et al., 2019). In contrast to pinacidil, diazoxide and NN414 attenuated morphine tolerance.

Diazoxide was examined in combination with analgesic drugs. In a model of PGE_2_-induced hyperalgesia, diazoxide reversed the antagonistic effect of NG-nitro L-arginine against diclofenac-induced antinociception (Alves et al., 2004a). Additionally, diazoxide potentiated the antinociceptive effect of fluoxetine against formalin-induced pain (Ghorbanzadeh et al., 2018). Combined with either dibutyrylguanosine cyclic monophosphate (db-cGMP) or sodium nitroprusside, diazoxide exerted marked antinociceptive effects against PGE_2_-induced pain (Alves et al., 2004b). Therefore, the analgesic properties of these drugs may be mediated via K_ATP_ channels. Injections of diclofenac, carbamazepine, or fluoxetine (Ghorbanzadeh et al., 2019; Ghorbanzadeh et al., 2018; Alves et al., 2004a), as well as KCOs (Du et al., 2011; Alves et al., 2004b), failed to elicit an antinociceptive effect when administered to contralateral paws in animals treated with pain inducers, indicating that the analgesic site of action is peripheral. Levcromakalim did not induce an analgesic effect (Christensen et al., 2022), which is discussed further below in relation to human trials.

### 4.6 Relation to human trials and future perspectives

Intraplantar administration of levcromakalim reportedly exerted no effect on mechanical withdrawal thresholds (Christensen et al., 2022), indicating that the hyperalgesic and allodynic effects of systemic administration (Christensen et al., 2022; Christensen et al., 2020) are not mediated via local activation of K_ATP_ channels in the paws. Hypersensitivity is likely to be mediated by vascular smooth muscle K_ATP_ channels (Christensen et al., 2022). These findings are consistent with trials conducted in healthy participants, where an intravenous infusion of levcromakalim reportedly induced headaches and dilated cephalic arteries (Al-Karagholi et al., 2019a); however, these effects were not observed when levcromakalim was administered intradermally or intramuscularly to the temporal muscle (Al-Karagholi et al., 2019b). Furthermore, all patients with migraine who received intravenous levcromakalim developed migraine attacks (Al-Karagholi et al., 2019c). These findings indicate that peripherally located K_ATP_ channels are unlikely to be sites of levcromakalim action. However, human data cannot be directly compared with rodent data because the measurement of pain is methodologically distinct, and interspecies differences in the molecular mechanisms underlying pain cannot be overlooked (Christensen et al., 2020). Data from human studies are inconsistent with the results of preclinical studies utilizing other KCOs; for example, systemic pinacidil was found to attenuate pain in a postoperative pain model in rodents (Shen et al., 2015; Qian et al., 2016; Cao et al., 2015), and diazoxide reportedly potentiates the antinociceptive effect of sildenafil (Vale et al., 2007) and alpha-2-adrenoceptor agonists (Galeotti et al., 1999b). Intraplantar (Du et al., 2011; Ghorbanzadeh et al., 2019; Ghorbanzadeh et al., 2018; Ortiz et al., 2002; Alves et al., 2004a; Alves et al., 2004b; Picolo et al., 2003; Sachs et al., 2004) or dorsal root ganglion (Du et al., 2014) administration of pinacidil and diazoxide attenuated locally induced pain in preclinical studies, in contrast to local administration of levcromakalim in humans (Al-Karagholi et al., 2019b) and animals (Christensen et al., 2022).

## 5 Conclusion

In preclinical studies, the opening of K_ATP_ channels attenuates induced pain and potentiates the effects of analgesic drugs at the spinal and supraspinal levels. The analgesic properties of cromakalim and the involvement of central K_ATP_ channel activity in pain modulation in diabetic populations warrant further investigation. Systemic and local administration of KCOs attenuates induced pain and potentiates the effects of analgesic drugs, except levcromakalim, which causes pain when administered systemically and has no effect on pain when administered locally. The effects of levcromakalim in preclinical investigations are consistent with the results observed in the human trials. Future studies should explore the differences in K_ATP_ channel activation between rodents and humans, as well as the differences in activation sites between levcromakalim and other KCOs.

## Data Availability

The original contributions presented in the study are included in the article/supplementary material, further inquiries can be directed to the corresponding author.

## Abbreviations

ATP, Adenosine triphosphate; BK, Bradykinin; CCI, Chronic constriction injury; DRG, Dorsal root ganglion; db-cGMP, Dibutyrylguanosine cyclic monophosphate; h, Hour; HS, Hypertonic saline; i.c.v., Intracerebroventricular; i.m., Intramuscular; i.p., Intraperitoneal; i.pl., Intraplantar; i.t., Intrathecal; i.v., Intravenous; K_ATP_, Adenosine triphosphate-sensitive potassium channels; KCL, Potassium chloride; KCO, K_ATP_ channel openers; L5, Fifth lumbar nerve; Min, Minutes; MWT, Mechanical withdrawal threshold; NN414, Tifenazoxide; PGE2, Prostaglandin E2; p.o., Peroral; R-PIA, N6-R-phenylisopropyladenosine; s.c., Subcutaneous; SMIR, Skin/muscle incision and retraction; SNL, Spinal nerve ligation; STZ, Streptozotocin; WT, Wild type.
